# Printable all-dielectric water-based absorber

**DOI:** 10.1038/s41598-018-32395-1

**Published:** 2018-09-27

**Authors:** Patrick J. Bradley, Max O. Munoz Torrico, Conor Brennan, Yang Hao

**Affiliations:** 1 School of Electronic Engineering and Computer Science, Queen Mary University of London, London, E1 4NS UK; 2 School of Electronic Engineering, Dublin City University, Dublin, D09 W6Y4 Ireland

## Abstract

The phase interplay between overlapping electric and magnetic dipoles of equal amplitude generated by exclusively alldielectric structures presents an intriguing paradigm in the manipulation of electromagnetic energy. Here, we offer a holistic implementation by proposing an additive manufacturing route and associated design principles that enable the programming and fabrication of synthetic multi-material microstructures. In turn, we compose, manufacture and experimentally validate the first demonstrable 3d printed all-dielectric electromagnetic broadband absorbers that point the way to circumventing the technical limitations of conventional metal-dielectric absorber configurations. One of the key innovations is to judicially distribute a dispersive soft matter with a high-dielectric constant, such as water, in a low-dielectric matrix to enhance wave absorption at a reduced length scale. In part, these results extend the promise of additive manufacturing and illustrate the power of topology optimisation to create carefully crafted magnetic and electric responses that are sure to find new applications across the electromagnetic spectrum.

## Introduction

The 3d shaping capabilities of additive manufacturing technologies have long been exploited in the fabrication of objects with unusual geometries^1^. The possibility of combining multiple materials into a single geometry is an even more tantalising asset that invariably adds an extra dimension to the available design space^2^. These concepts open the way towards the manufacturing of functional heterogeneous materials that potentially enables the realisation of enticing functionalities into 3d objects. From an electromagnetic perspective, additive manufacturing (AM) research is broadly divided along two delineations; the development of composite 3d printable dielectrics^3^ and; the imposition of converting transformation optics (TO) inspired metamaterial designs into 3d amenable structures which are often inconsistent with AM ideology^4–6^. In all cases, to date, no research has genuinely embraced the AM concept and developed holistic design frameworks that facilitate the formation of 3d printable EM devices directly. Such aspirations required us to explore alternative design methods and materials which can concurrently balance manufacturing sensitivities with the utility of design. We propose an additive manufacturing route and associated design principles that enable programming and fabrication of synthetic multi-material microstructures. In turn, these can independently tailor their electric and magnetic response to incident radiation, capabilities central to the metamaterial mantra. The inference here is that our solution must be binary machinable all-dielectric compositions, that when suitably designed manifest the same functionality of resonant metal-based metamaterial architecture while exhibiting none of their deficiencies^7–9^.

A promising pathway to securing a robust diamagnetic response to match its electric counterpart is to use high-index subwavelength dielectric particles which have been shown to support robust magnetic and electric dipolar resonance^10,11^. Empowered with the Huygens’ principle^12,13^, the proposed structure provides potential solutions to the design of efficient single-layered metamaterials and our interpretation thereof, a perfect wideband absorber. The underlying principle is that if a particle exhibits a critical combination of spectrally overlapping pure magnetic and electric dipolar resonances of equal strength, arbitrary control over the scattering field can be guaranteed under plane wave illumination. In particular, the direction of this scattered field is solely dependent on the phase relationships of each particle^14,15^.

In this work, we experimentally verify this approach by demonstrating a 3d printed perfect electromagnetic absorber that is impedance matched over a wide range of frequencies paving the way to circumventing fundamental limits of conventional metal-dielectric configurations. A keys innovation is the facilitation through topology optimisation of a dispersive soft matter with a high-dielectric constant, water, within a 3d printed low-dielectric matrix to create a tailored EM response. This concept will open up possibilities to add further functionality to metamaterial and meta-surface^16,17^ designs aided with the ever-increasing capabilities in additive manufacturing, the limiting factor been the direct availability of high-dielectric printable/substitutable materials. Currently, polyjet printable high-index dielectrics do not commercially exist, although some efforts have been made to create fused deposition printable BaTiO3/ABS polymer composites with permittivities of up to 8^3^.

Contrary to traditional applications of Kerker’s first condition^18^, that of an impedance matched material corresponding to an ideal Huygens secondary source, we embrace the often-overlooked loss component and focus on developing a mechanism by which we can decouple the impedance matching requirement from dispersive material responses. Through the deliberate distribution of the fluid within the structure, we can induce specific electric and magnetic dipole behaviour. Specifically, the incident field is efficiently coupled into the water without reflection at the interface, ensuring complete absorption of the EM energy. We independently control gradual sizes and location of water droplets within the host structure ensure that multiple enhanced electric and magnetic resonances are overlapping, achieving wideband frequency responses. These properties have not yet been accomplished and are not possible from conventional homogeneous architectures alone or single core-shell compositions^19^.

Although water is chosen in this design, the proposed approach is a general one, highlighting the potency of AM and high-dielectric materials to synthesise multi-material single-layered metamaterials than can produce complex tailored electromagnetic responses. Indeed, for the given design water is a natural choice providing the required functionality while been volume preserving, thermally tunable, optically transparent, abundant, bio-compatible and switchable^20^. As an all-dielectric structure, the proposed approach will work for high power microwaves, where absorption devices with metallic components are problematic due to arcing^21^, is also feasible. Other practical implementations that would benefit from this technology include optically transparent EM absorbers and electromagnetic shielding that requires air cooling, a by-product of the design. It should be noted that although the 2d z-invariant design proposed here could be fabricated using subtractive manufacturing or injection moulding due to the generalism of the design routine AM provides the greatest fabrication flexibility as well as design-on-demand functionality.

## Discussion

**Figure 1:**
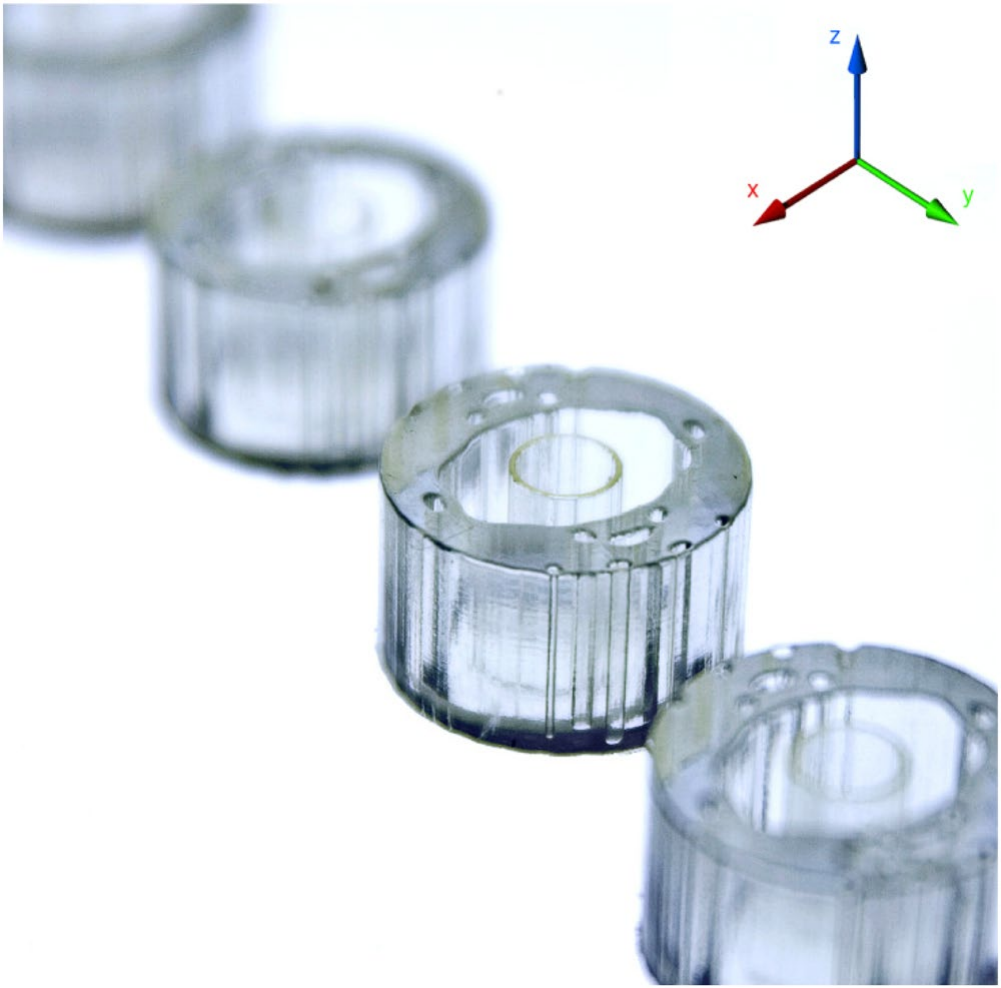
The three-dimensional printed structure as per experimental set-up manufactured with Stratasys VeroClear Fullcure 810 transparent photopolymer (VC810) in HQ gloss mode on the Objet30 Prime printer to a height of 13 mm.

**Figure 2:**
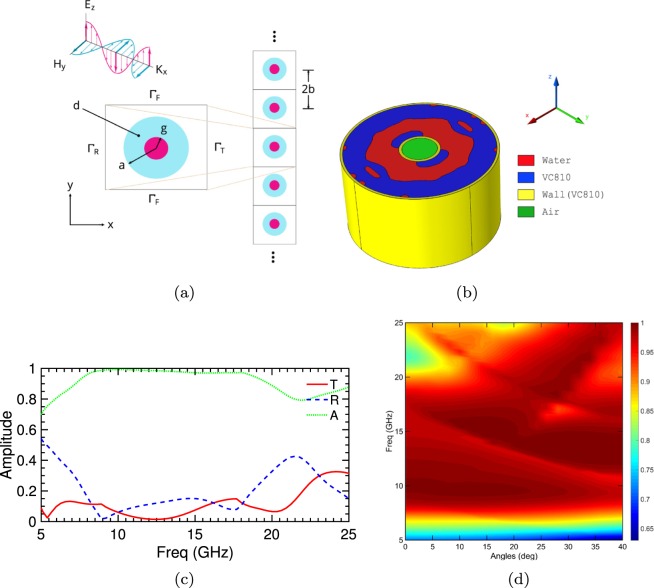
Broadband all-dielectric symmetric absorber geometry and simulation set-up with corresponding validating simulation results. (**a**) Design domain d and boundary conditions for periodic cylindrical structures illuminated by a normally incident plane transverse electric wave propagating in the x-direction. The upper and lower boundaries Γ_*F*_ are set as Floquet periodicity with Γ_*R*_, Γ_*T*_ representing the reflection and transmission boundaries respectively. *g* = 3.17 *mm* denotes the radius of the internal core, the thickness of the design space domain is equal to (*a* − *g* = 8.59 *mm*), and the distance between the centres of successive cylinders is 2*b* = 34.32 *mm*. (**b**) The geometry and makeup of a single unit cell within the one-dimensional infinite array, wall (yellow) thickness is 450 *μm*, the outer and inner diameter of the design space (blue) is given by *a* and *g*, respectively. (**c**) Transmission (red *T*), reflection (blue *R*), and absorption amplitudes (green *A* = 1 − | *T*|^2^ − |*R*|^2^) indicating near-perfect absorption from 8–18 GHz under normal illumination. (**d**) Absorption as a function of plane wave incident angle indicating 90%^+^ absorption over the bandwidth 8–18 GHz for 0–40 degrees.

Our analysis starts with the theoretical conditions required for total absorption of normally incident plane waves by an infinite 1d periodic array of fictitious electric and magnetic dipoles as established by the antenna theory^22,23^. This array is composed of unit elements with subwavelength period *α* that under electromagnetic illumination induce an electric ***p*** and magnetic dipole moment ***m***. These moments are mutually orthogonal with their corresponding components aligned along the electric and magnetic incident field directions. As prescribed by Huygens’ principle this array of moments can create symmetrically radiating secondary plane waves of equal amplitude in the transmitted and reflected directions^23,24^, which can be expressed by

1 $$\begin{array}{rcl}{E}_{z,t}(x) & = & {E}_{z,inc}(x)+{E}_{z,s}(x)=\exp (\,-\,i{k}_{0}x)+\frac{i{k}_{0}{\zeta }_{0}}{2\alpha }({m}_{y}-c{p}_{z})\exp (\,-\,i{k}_{0}x)\\  & = & {E}_{z,inc}-\frac{1}{2}{E}_{z,inc}-\frac{1}{2}{E}_{z,inc}\mathrm{=0}\\ {E}_{z,r}(x) & = & \frac{i{k}_{0}{\zeta }_{0}}{2\alpha }(-{m}_{y}-c{p}_{z})\exp (\,-\,i{k}_{0}x)=-\frac{1}{2}{E}_{z,inc}+\frac{1}{2}{E}_{z,inc}=0\\ {\rm{if}}\,c{p}_{z} & = & -\,{m}_{y}=\frac{\alpha }{i{k}_{0}{\zeta }_{0}}\end{array}$$

where *c*, *ζ*_0_, *k*_0_ is the speed of light, free space wave impedance and wavenumber respectively. The ensuing interference pattern between the collective magneto-electric response of the metamaterial and the incident plane wave will determine the extent of the absorption. Consequently, when the magnetic and electric dipole scattered response are both equal to negative one-half of the incident wave amplitude and resonates at the same frequency with equal width, the result will be a zero transmission and reflection composition (Supplementary Fig. 4(a,b)). As should be clear by this analysis, the amplitude of combined out-of-phase dipole resonance in the forward direction is zero. The negative interference eliminates the electric field component of the incident plane wave with the combined effect of the two dipole resonances on the transmit side. One further prerequisite, not discussed here but which should be evident is that for total symmetric absorption, all cross-coupling polarisability coefficients must vanish^23^. Such a design leads to reciprocal electromagnetic responses for illumination from either side of the absorber without the need for a coherent source^25^. In classical electrodynamics, this arrangement of balanced electric and magnetic dipoles is known as a Huygens’ pair, and it is conceptually equivalent in volumetric terms to a matched impedance material {*ε*_*r*_ = *μ*_*r*_} with loss component carefully chosen to ensure zero transmission. Notably, there is far more design freedom for accomplishing this arrangement beyond traditionally investigated simple single or multi-core metallic/dielectric compositions with the precipitation that such capacity is not achievable with dielectric materials alone. To this end, we show that such configurations are possible through the synthesises of all-dielectric multi-material structures by realising an EM absorber that suppresses all reflections and transmissions. We back up these ascertains through numerical simulations, extracting equivalent dipole moments models^22,23^, extracted effective material coefficients^26^ and through experimental validations. The resultant AM manufactured structure is shown in Figs 1 and 2(a,b); that of a 1d infinite array of dielectric inhomogeneous rods positioned along the y-axis with their central axis parallel to *z* with periodicity 2*b* and outer radius *a* under illumination by a normal incident plane wave in *x* with a *z*-polarised electric field *E*_*z*,*inc*_(*x*).

Transmission, reflection and absorption coefficients as a function of incident angle and wavelength are presented in Fig. 2(c,d); where an absorption coefficient of 1 corresponds to complete absorption. Together, they represent the success of the proposed structure in creating an all-dielectric wideband (8–18 GHz), angular independent (*θ* = 0–40 degrees) device with 90 % plus absorption for TM polarisation. Within this range, transmission and reflection amplitudes of −20 dB can be achieved with up to 98 % absorption. Based on a generalisation of Kerker’s conditions^18,19,27,28^ we can recast Eq. 1 for off-angles radiation suppression as

2 $$c{p}_{z}={m}_{y}{\cos }^{2}\,(\theta )-{m}_{x}{\sin }^{2}(\theta )=\frac{\alpha }{i{k}_{0}{\zeta }_{0}}$$

where the minus sign of the *y* component has been absorbed by the cross product (see Eq. 9). Thus the radiation can be suppressed for angles in the backward direction up to |*θ *− *θ*_*i*_| ≤ *π*/2 if this condition is adhered to (see Supplementary Fig. 10(a–d)). The source of angular stability is a direct consequence of both the enforced 4-way rotational symmetry (Supplementary Fig. 2(a)) and the wide-angle excitation of the magnetic response due to the water distribution as evident in Supplementary Fig. 11(a–d).

**Figure 3:**
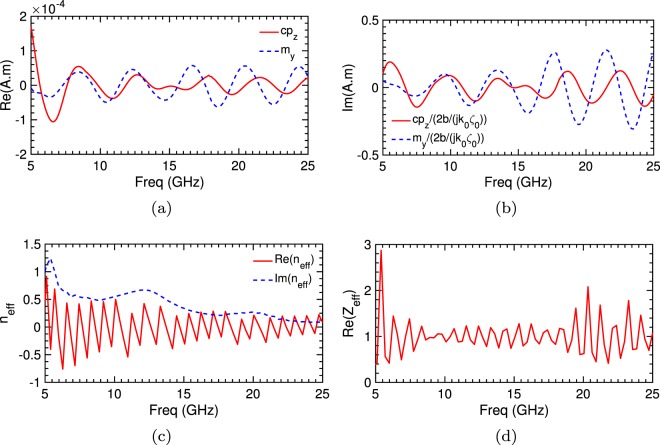
Simulated extracted equivalent moment and dielectric properties for absorber design. (**a**) Real and (**b**) scaled imaginary component of the electric (red) and magnetic (blue) dipole moment resonances, where a zero response fulfils the theoretical case for perfect absorption. Real and imaginary parts of the (**c**) inverted effective refractive index and (**d**) impedance, extracted from S-parameters for normally incident plane wave. The structure has a near-zero refractive index (indicating zero phase evolution), impedance near-unity and large imaginary part over the optimisation band of interest. This configuration,therefore, corresponds to a medium that is impedance matched ensuring a low reflection interface (no reflections) with high loss, where electromagnetic waves are strongly evanescent (no transmission).

The inclusion of water and the robustness of our design are both demonstrated in (Supplementary Fig. 3(b)). When the water is completely removed, the absorption capability is eliminated; while substituted by materials with equally high but non-dispersive permittivity, a minimal effect is observed. This following result is to be expected as the mandatory phase interlock between the electric and magnetic dipole response would be relatively unaffected by small amplitude changes in the permittivity. Now we explore the origin of wave absorption from the proposed structure first by focusing on extracting dipole moments^22,23^ derived from the polarisation currents **J** excited inside the volume (see methods). In Fig. 3(a,b), the real and imaginary component of the electric and magnetic moments as defined in methods are extracted and plotted, where a zero response fulfils the ideal case for complete absorption (Eq. 1). Physically this requires the electric and magnetic moments to be balanced, in phase and be purely imaginary (resonant). Although with some difference, the general trend observed strongly resembles these criteria in the frequency band of interest (8–18 GHz), followed by a breakdown after that. Quadrupole responses are negligible for this excitation (see methods), reinforcing the reliability of the dipole model to indicate that the suppression of reflection and transmission from our array originates from the interference between the induced electric and magnetic dipoles.

Let us now examine the effective refractive index *n* and wave impedance *Z* extracted from the simulated reflection and transmission coefficients. These offer additional insights into the working of our structure that are not evident from the dipole moments alone. These results were self-consistent with what we expected from the Multipole expansion and do provide a unique characterisation of the structure, verified by comparisons with multiple layered structures. The recovered dielectric constants as shown in Fig. 3(c) indicate a near-zero refractive index that oscillates between +0.5 to −0.5 a consequence of the transmission data close to zero with a corresponding imaginary part of approximately +0.5. There is thus a near-zero phase evolution inside the structure, alternating between a negative and positive relative phase. In turn, the wave travelling through the highly dispersive medium experiences strong evanescence resulting in a near-zero transmission on the exit side. With the entirety of the energy impinging on the structure coupled into the interior, as proven by Fig. 3(d) which illustrates an impedance of approximately 1, complete absorption is thus accomplished. One can observe these effects in (Supplementary Fig. 6(a,b)), where the cylindrical array is replaced with the equivalent extracted material, (Supplementary Fig. 5(a,b)), for the electric field measured along the x-axis at y = 0 for 10 GHz. At normal incidence, the near-unity incident and near-zero transmission along the axis is observed. The predicted destructive interference (Eqn. 1) manifested itself as a *π*-path delay in the forward direction between the incident and scattered fields of equal amplitude ensuring the required zero transmission. In all cases breakdown in performance is visible outside the band of interest at 8–18 GHz, with the phase of the electric and magnetic dipole losing cohesion (Fig. 3(a,b)), reducing loss Fig. 3(c) and impedance mismatching Fig. 3(d).

**Figure 4:**
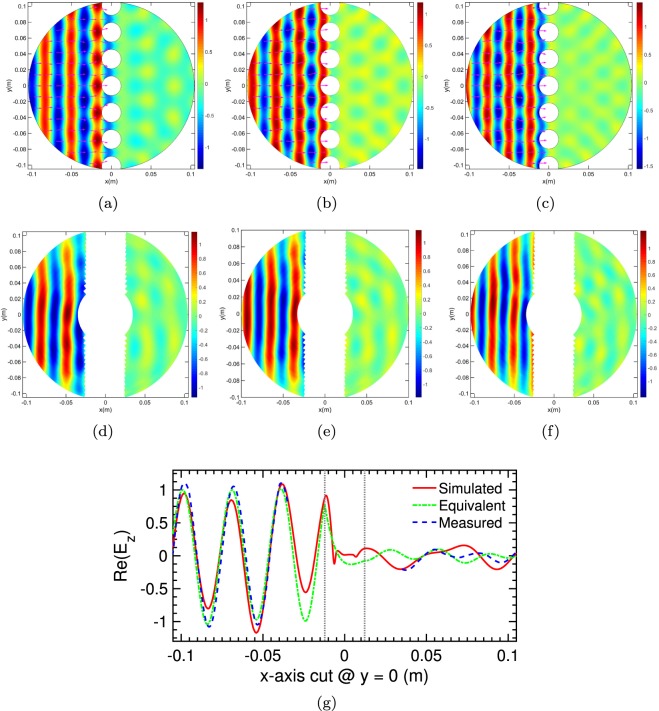
Simulated and measured real component of total electric field, z component (V/m), normalised to free space at varying frequencies and incident angle for 7 cylindrical absorbers periodical arranged along the y-axis @ x = 0. (**a**–**c**) Simulated electric field and power flow time average arrow plot for 9, 10, 11 GHz respectively for a normally incident plane wave. (**d**–**f**) Associated measured results for the same set-up, note: due to the rotary nature of the probing apparatus and the fact no data points could be probed in the vicinity of the samples, a white-zone between the two data sets exists. (**g**) Comparison plot of the total electric field from along the x-axis @ y = 0 for 10 GHz, where grey lines indicate boundary of the medium, for simulation (red) vs measured (blue) vs inverted effective medium (green) data. The proposed structure has a near-unity electric field in the reverse direction and near-zero field in the transmission side validating our case for near-perfect absorption. Based on the calculated standing wave ratio (SWR) along these cuts the reflectivity magnitude for the simulated, equivalent and measured are 0.037, 0.0124 and 0.0115 respectively where theoretically 1 indicated complete reflection and 0 correspondingly, no reflection. Therein equivalent refers to a homogeneous structure composed of the effective material coefficients extracted from the simulated reflection and transmission coefficients.

To experimentally demonstrate the absorption capabilities of our proposed design; an array of 7 cylinders with a height of 13 mm have been fabricated through a three-dimensionally printing process as per Figs 1 and 2(b). A distribution of electric field as a function of position was performed using an in-house built planar parallel waveguide chamber (see Methods for details). The simulated and measured real part of the total electric field, z component, are all normalised to free space measurements with results limited to frequencies in the X-band. There is a remarkable overall agreement between the simulated and measured results, Fig. 4(a–f), which demonstrates the absorption capabilities of the proposed device. All experimental data exhibit excellent signal-to-noise ratios, and the collimating effect of the lens is evident as the source of a plane-wave incident with only a slight deviation in the amplitude as the beam moves away from the x-axis. Correspondingly, the phase front is remarkably uniform, Fig. 4(g) summarises these results for 10 GHz, comparing the total electric field from unit cell simulation, effective media simulation and experimental data along the x-axis at y = 0. The agreement is excellent with a near-unity electric field in the reverse direction and near-zero field on the transmission side, validating our prediction for near-perfect absorption. There is a slight mismatch on the reflection side close to the boundary interface for the effective medium result, but this is to be expected from any abridged model. Based on the calculated standing wave ratio (SWR) along these cuts the reflection coefficient magnitude for the simulated, effective medium and measured data are 0.037, 0.0124 and 0.0115 respectively where 1 would indicate complete reflection and 0, no reflection. To complete the picture, simulated and measured field maps for 45-degree incidence are shown in (Supplementary Fig. 8(a,b)) which illustrate that the proposed absorber is angular independent as shown in Fig. 2(d).

**Figure 5:**
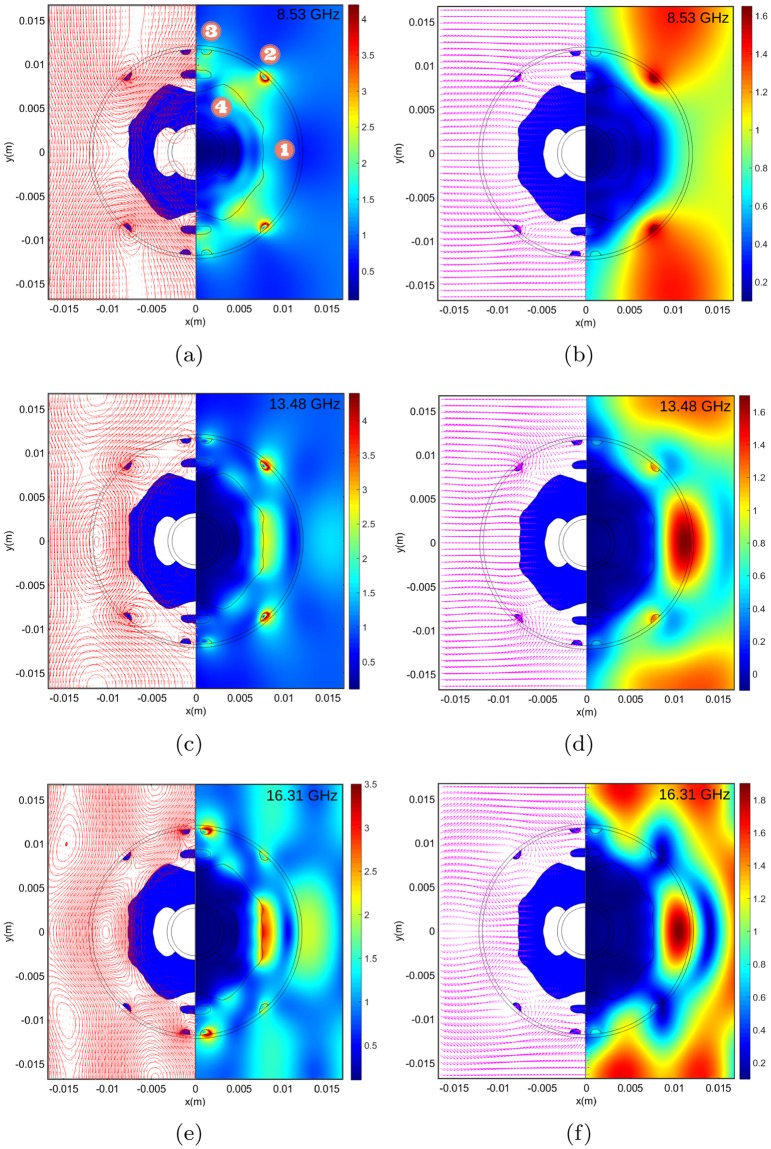
Simulated surface magnetic, power flow time average and electric field amplitude plots normalised to free space for three discrete frequencies which correspond to key electric and magnetic surface maximum resonances. (**a**,**c**,**e)** −x illustrates logarithmic scaled surface arrow & streamlines (red) for the *x* and *y* magnetic field components at 8.53, 13.48 and 16.31 GHz respectively for illumination from LH boundary (blue represent areas of water), +x 180° rotated image of the corresponding magnetic field magnitude. (**b**,**d**,**f**) −x illustrates related logarithmic scaled power flow time average arrow (magenta) and +x the 180° rotated electric field magnitudes. An evolution in the mode centres is evident as the wavelength decreases and penetrates deeper into the space between the periodic cylinders. Individually they evolve from position 2 at 8.53 GHz to positions 1 & 2 at 13.48 GHz to finally 1 & 3 at 16.31 GHz.

As a final measure, we examine the electric and magnetic surface field maxima for three fundamental frequencies (see Supplementary Fig. 9(a)) within the band 8–18 GHz, Fig. 5(a–f). As expected, the fields are highly confined to the regions of high permittivity, with a high degree of symmetry in the response. The Poynting vector (power flow time average arrow plots) offer real insight into the complex interactional effect of this electric and magnetic enhancement, illustrating in detail the direction of the energy flow in and around the device. In all cases, the enhanced magnetic and electric dipole moments effect is such that the power flow is directed into the central water reservoir (position **4**) where dissipation of the impending energy ensures zero fields evident at the cylinder centre (Supplementary Fig. 9(b)) and consequently no leakage in the forward direction. It is clear that the enhanced electric and magnetic activity at the interface of the water medium ensures wave impedance matching and consequently zero reflections. An evolution in the mode centres is also evident as the wavelength decreases and penetrates deeper into the space between the periodic cylinders. Individually they evolve from position **2** at 8.53 GHz to positions **1** & **2** at 13.48 GHz to finally **1** & **3** at 16.31 GHz (see the supporting videos in Supplementary for illustration this evolution in more detail). It is worth mentioning the that these electric and magnetic enhancements are broadly in line with the dipole model maxima (see Fig. 3(a,b). Rather interestingly, the same modal positions for 16.21 GHz play a pivotal role in the absorption characteristics for 20 and 40-degree illuminations at 13.48 GHz (see Supplementary Fig. 11(a–d)) albeit without the same symmetry.

The success of the proposed approach is compelling given the effectiveness with which it utilises existing additive manufacturing technologies to synthesise all-dielectric multi-material structures that can produce complex tailored electromagnetic responses. Additionally, these results bring into question the commonly held viewpoint that AM role in electromagnetic is limited to just replacing existing manufacturing approach, when in fact if truly embraced AM can be the catalysts for creating enhanced real-world EM devices by adding an extra dimension to the design process. Indeed our use of unorthodox materials coupled with a re-imagining of Huygens’ principle will serve to drive further technology breakthroughs across the electromagnetic spectrum by using similarly inspired design methodologies.

## Methods

### Experimental

To obtain the field maps presented here, we assembled a planar parallel waveguide chamber consisting of an upper suspended and lower rotational plate (1 m diameter 5 mm thick aluminium) mounted on a CPU controlled step motor with X-band capabilities (8–12 GHz). The height of the waveguide is 13 mm (along the z-axis), which supports the dominant TEM mode only, as all other modes are evanescent at frequencies below the lowest cut-off frequency, *f*_*c*_ = *c*/(2*d*) (d the spacing between the two plates). For this mode the electric and magnetic fields do not vary between the plates, this invariance ensures that a map of the fields at any plane along z within the chamber should provide an equivalent characterisation of the scattering. To replicate the conditions of an infinite waveguide in the x-y plane, effective suppressing of any reflections is required at the boundaries of the plates. This is facilitated by terminating the mapping area in all directions with tapered saw-tooth patterned foam microwave absorbers. Excitation of the chamber is achieved by an x-band rectangular waveguide adapter, at its phase centre, a line-source-equivalence can be assumed that acts as the epicentre of a cylindrical wavefront. To emulate a plane wave incident field, a single-surface conversion lens is placed at 10 mm from the waveguide aperture^29^. To ensure the ray exiting the lens are parallel, the second surface is planar, coinciding with the eikonal nature of the emergent wave. The collimated portion of the feed radiation is limited by the asymptotes of the hyperbola. Thus we narrow the lens edge angles to 40 degrees. Subject to this restriction and the availability of a material with refractive index of 1.63, a three-dimensional printed lens was designed and manufactured using a Vero Black Fullcure 870 photopolymer (*ε*_*r*_ = 2.661 + 0.0071*i*, measured using an Agilent split cylinder cavity resonator (85072 A) operating at 10 GHz). With the inclusion of the converter lens, the scanning area is reduced to a maximum radius of 105 mm. The fields external to the samples in the chamber are detected by a coaxial antenna mounted on the upper place with its centre pin extended by 5 mm into the chamber and dielectric sheath cut level with the plate. While the coaxial centre pin could have been mounted, flush to ensure mapping of the fields internal to our samples, the considerable reduction in signal level could not be justified, as our primary consideration was the fields external to the samples. A Vector Network Analyser (VNA) (Agilent N54244A) serves as both the microwave source through the waveguide and detection of the return signal via the coaxial antenna as a complex transmitted S-parameter S21. As a consequence of the z-axis translational invariance, the EM modes of interest correspond to solutions of the two-dimensional scale wave equation for a z-polarised electric field. Thus, the measured S21 reflects the corresponding local electric field *E*_*z*_ directly^30^. Measurements were probed at 5 mm radial increments subject to a 0.12° angular resolution and subsequently interpolated using a linear function to generate the field maps. A custom LabView program coordinated the motion of the platform and the record-hold data acquisition of the VNA. To confirm the validity of measurement set-up, we acquire the complex electric field as a function of position for an empty chamber and plot its normalised real component and phase (Supplementary Fig. 7(a,b)). Both plots exhibit excellent signal-to-noise ratios, with the collimating effect of the lens evident. Also, the phase front is uniform and in very close accord with what was expected.

### Numerical Implementation

The design problem is prescribed as a two-dimensional periodic scenario, Fig. 2(a), and is formulated as an optimisation problem to determine the material distribution in the domain *d*, with COMSOL Finite Element Method solving for both the equilibrium and adjoint equations. Therein, the upper and lower boundaries Γ_*F*_ are set with Floquet periodicity, and the unit cell is illuminated by a normally incident plane transverse electric wave propagating in the x-direction with Γ_*R*_,Γ_*T*_ representing the reflected and transmitted boundaries respectively. While the criteria for complete absorption has been set, we must now determine effective constraints and geometric inequalities that will seed this optimisation problem. A natural and rather well-defined approach is to adopt the simple structure of an infinite array of dielectric insulating homogeneous perfect electrically conducting (PEC) circular rods as our starting position. Such configurations have representations analytically for the transverse electric scattered fields based on Schlomilch series at arbitrary incident angles^31^. It has been shown^19^ following this procedure that analytical conditions for perfect absorption can be established that provide robust inequalities to govern these compositions {0.65 < *a*/*n* < 0.75,0.2 < *g*/*a* < 0.35} with the period of the array given a maximum value of *b* ≅ *λ*_0_/2 and based on permittivities of similar real and imaginary parts $\{3.5 < \Re [{\varepsilon }_{r}] < 5,-\,5\Im [{\varepsilon }_{r}] < -\,1.5\}$. Such constraints limit the potential of designing wideband printable absorbers, however; they do serve a useful function of minimising the set of the unknown within the optimisation problem. Following these inequalities the results of a primary line search provide an operational frequency of {*f*_*α*_ = 8.73 *GHz*} based on the selected dimensional ratios of {*b* = *λ*_α_*0.49, *a* = b*0.7 and *g* = 0.27*a}. An external wall thickness of 450 *μm* is assumed for an maximum outer radius of 12.22 *mm* (*λ*_*α*_/2.8), *g* = 3.17 *mm* and periodicity of *λ*_*α*_ with a mesh density of *λ*_*max*_/8 applied. To ensure symmetric and angular independent absorption four-fold symmetry restrictions are imposed on the design domain *d* as per Fig. 2(a), by enforcing a 90, 180 and 270 degree clockwise rotation transformation on the projected filtered density value $\bar{\rho }(\rho )$ at each iteration for a single quadrant (Supplementary Fig. 2(a)).

An essential and characteristic aspect of this optimisation is that the internal architecture of the cylindrical element mainly modify the way the enhancement is realised since the distribution of the electromagnetic field in the material plays a crucial role. Besides, governance over the quantification of the dissipation of electromagnetic energy within the structure is solely dependent on the allocation of the water within this matrix. As such, the primary focus of the optimisation sequence is the deliberate distribution of water within a dielectric matrix. In this we use the commercially available Stratasys VeroClear Fullcure 810 transparent photopolymer (VC810) but as stated earlier any low contrast printable dielectric will work. The permittivity profile of VC810 is confirmed using an Agilent split cylinder cavity resonator (85072 A) operating at 10 GHz given us an average reading of *ε*_*r*_ = 2.67 + 0.0075*i*. With the correct cost function multiple spectrally overlapping enhanced electric and magnetic resonances can be induced of equal strength within the sub-wavelength unit cell, guaranteeing the wideband all-dielectric absorption response. Accordingly, we define the cost function as minimising the sum of the squares for the reflection and transmission coefficients over a suitable bandwidth

3 $${\rm{minimise}}:\,{\rm{cost}}(\rho )=\sum _{i\in {{\mathbb{N}}}_{f}}\,(|S{\mathrm{11|}}_{i}^{2}+|S{\mathrm{21|}}_{i}^{2})\,\,\,8\le f\le 18\,{\rm{GHz}}\,\,\,{{\mathbb{N}}}_{f}=25$$

4 $${\rm{subject}}\,{\rm{to}}:\,{\rm{g}}=\sum _{m\in {{\mathbb{N}}}_{e}}\,{\rho }_{m}{\nu }_{m}\le {V}_{c}\,\,\,0\le {\rho }_{m}\le 1\,\forall m\in {{\mathbb{N}}}_{e}\,\,\,{{\mathbb{N}}}_{e}=1741$$

where ${{\mathbb{N}}}_{f},{{\mathbb{N}}}_{e}$ are the index set of all frequencies and finite element discretisation elements respectably, as defined within the space *d* between the outer and inner walls as shown in Fig. 2(a). While *V*_*c*_, *v*_*m*_ express the total volume which can be occupied by water and the volume of individual FEM elements respectively. Topology optimisation (TO)^32^ subsequently provides the systematic approach required to account for this complex environment in the design process, yielding unintuitive designs by judicially and iteratively re-distributing material within design bounds, optimally (see Figs 1 and 2(b) for final design). In essence, the TO replaces the optimisation problem with a material distribution problem within the architecture using a characteristic density function *ε*_*r*,*m*_ given by

5 $$\begin{array}{rcl}{\varepsilon }_{r,m}(\bar{\rho }(\rho )),\omega ) & = & \Re ({\varepsilon }_{r,a})+{\bar{\rho }}_{m}{(\rho )}^{p}(\Re ({\varepsilon }_{r,w}(\omega ,{T}_{w}))-\Re ({\varepsilon }_{r,a}))\\  &  & -\,i[\Im ({\varepsilon }_{r,a})+{\bar{\rho }}_{m}{(\rho )}^{p}(\Im ({\varepsilon }_{r,w}(\omega ,{T}_{w}))-\Im ({\varepsilon }_{r,a}))]\end{array}$$

that defines the material for each destination element *m* within a FEM discretisation. Therein *ε*_*r*,*a*_ is the permittivity of the solid phase VC810 and *p* is the power factor that penalises intermediate densities^33^. *ε*_*r*,*w*_(*ω*, *T*_*w*_) is the frequency and temperature dependent permittivity of water as defined by the Debye formulation^34^ analytical implemented in COMSOL and plotted in (Supplementary Fig. 3(a)) for a water temperature set to 19 °*C*. A fictitious porous material with density, *ρ*_*m*_, is required to allow a continuous transition between the two material phases which can assume values from 0 to 1. While this method may seem contrary to manufacturing constraints, it is necessary to ensure a flexible optimisation approach that can guarantee an optimised layout. Without some form of regularisation, the topology optimisation problem will invariably become dependent on the FEM mesh composition and resolution. This can set limits on the size of details within the topology solution and start to influence the solution itself by directing it towards irregular artefact or other mesh-dependent pathologies which in turn can compromise the reliability of the final design. To regularise the scalar function, we introduce a smoothing function that neutralises these anomalies which are subsequently projected towards a binary solution to arrive at the physical density function ${\bar{\rho }}_{m}(\rho )$ of an element *m*. Updating of the densities is streamlined by the incorporation of a sensitivity analysis based on the adjoint variable method, ensuring high-resolution problems are obtained in a computationally efficient manner. To ensure seamless, continuous optimisation within the COMSOL environment, we employ a coupled Helmholtz partial differential equation^35^ as our mesh independent filtering solution

6 $$-{r}^{2}{\nabla }^{2}{\tilde{\rho }}_{m}+{\tilde{\rho }}_{m}={\rho }_{m}$$

This equation implicitly performs our filtering, acting as a low-pass filter that operates on the raw scalar function *ρ*, to produce the smoothed scalar function $\tilde{\rho }$. The scalar length parameter *r* is set to the maximum mesh size and manipulates the profile of the monotonically decaying averaging integral centred at the source cell, in effect introducing a minimum length scale into the design. As this parameter approaches zero, we see an increase in isolated peaks until the function approaches that of Dirac delta function and the output field resembles that of an unfiltered one (Supplementary Fig. 2(d)). Therein, the diffusion coefficient *c* is set to *r*^2^ and the source term to *ρ*, with zero flux imposed on the design perimeter consistent with homogeneous Neumann boundary conditions. To arrive at a binary solution and by extension, a printable one, while accommodating gradient-based optimisation that requires a continuous, differentiable characteristic function, careful modification of the imposed intermediate densities $\tilde{\rho }$ is required. This balance can be achieved by implementing the volume-preserving nonlinear Heaviside filter^36^, taking the form of an analytical function with corresponding derivatives given by

7 $$\bar{\rho }(\rho )=(\begin{array}{ll}\eta [{e}^{-\beta (1-\tilde{\rho }/\eta )}-(1-\tilde{\rho }/\eta ){e}^{-\beta }] & {\rm{0}}\le \tilde{\rho }\le {\rm{\eta }}\mathrm{.}\\ (1-\eta )[1-{e}^{-\beta (\tilde{\rho }-\eta )/(1-\eta )}+(\tilde{\rho }-\eta )/(1-\eta ){e}^{-\beta }]+\eta  & {\rm{\eta }} < \tilde{\rho }\le 1.\end{array}$$

8 $$\frac{\partial \bar{\rho }(\rho )}{\partial \rho }=(\begin{array}{ll}\beta {e}^{-\beta (1-\tilde{\rho }/\eta )}+{e}^{-\beta } & {\rm{0}}\le \tilde{\rho }\le {\rm{\eta }}\mathrm{.}\\ \beta {e}^{-\beta (\tilde{\rho }-\eta )/(1-\eta )}+{e}^{-\beta } & {\rm{\eta }} < \tilde{\rho }\le {\rm{1.}}\end{array}$$

To satisfy volume preservation this approach introduces two scalar control entities, a projection parameter *β* which controls the smoothness of the Heaviside filter and *η* which indicates the position of the inflexion point of the projection. In effect, this means that all filtered values $\tilde{\rho }$ above a threshold *η* are projected, as a function of *β*, towards 1 and values below to 0. Practically, *β* is gradually increased on a periodic or conditional basis until such point that convergence has been met or a viable solution has been recovered (Supplementary Figs 1(a–d) and 2(b,c)). In this case, the projection control entity *β* is initiated at 0.125 and doubled ever 50 iterations to a maximum value of 256, with the inflexion point *η* equal to 0.5. This ensures that intermediate values are not penalised at early stages of the optimisation and that the topology can radically change. As the projection progressively increases, intermediate values are aggressively sanctioned, significantly reducing any topology changes, ensuring that a final binary solution starts to emerge. To safeguard the containment of the fluid within the dielectric matrix a volume fraction constraint of {*V*_*c*_ = 75%} is imposed to define the allowable fraction of the total volume *V*_*c*_ which can be occupied by water. A solution of the optimisation problem can now be obtained through any nonlinear programming algorithm, but it has been shown that the method of moving asymptotes (MMA) is ultimately best served to solve the topology styled optimisation problem^32^.

### Multipole expressions

To demonstrate the theoretical conditions for perfect absorption as set-out in Eq. 1, we replace the induced polarisation currents **J** excited inside the cylindrical volume with electric and magnetic multipoles that act as equivalent point sources for the scattered field in free space. From multipole theory^37,38^, the per-unit-length electric dipole **p**, magnetic dipole **m**, electric quadrupole **Q**_**e**_ and magnetic quadrupole **Q**_**m**_ moments for the two-dimensional case can be obtained via integrating the induced current over the cross-section surface (S)

9 $${\bf{p}}=\int {\int }_{S}{\varepsilon }_{0}({\varepsilon }_{r}-1){\bf{E}}dS=\frac{i}{2\pi f}\int {\int }_{S}{\bf{J}}dS\,{\bf{m}}=-\frac{i\omega }{2}\int {\int }_{S}{\varepsilon }_{0}({\varepsilon }_{r}-1)({\bf{r}}\times {\bf{E}})dS=\frac{1}{2}\int {\int }_{S}{\bf{r}}\times {\bf{J}}dS$$

10 $${{\bf{Q}}}_{{\bf{e}}}=\frac{i}{2\pi f}\int {\int }_{S}({\bf{J}}\otimes {\bf{r}}+{\bf{r}}\otimes {\bf{J}})dS=\frac{i}{2\pi f}[\begin{array}{ccc}0 & 0 & -2{m}_{y}\\ 0 & 0 & 2{m}_{x}\\ -2{m}_{y} & 2{m}_{x} & 0\end{array}]$$

11 $${{\bf{Q}}}_{{\bf{m}}}=\frac{1}{3}\int {\int }_{S}(({\bf{r}}\times {\bf{J}})\otimes {\bf{r}}+{\bf{r}}\otimes ({\bf{r}}\times {\bf{J}}))dS=\frac{1}{3}\int {\int }_{S}[\begin{array}{ccc}2{r}_{x}{r}_{y}{J}_{z} & ({r}_{y}^{2}-{r}_{x}^{2}){J}_{z} & 0\\ ({r}_{y}^{2}-{r}_{x}^{2}){J}_{z} & -2{r}_{x}{r}_{y}{J}_{z} & 0\\ 0 & 0 & 0\end{array}]$$

where **r** is the observation vector in the cylindrical coordinates and the indices specify the vector component along a particular spatial direction. It should be noted, that depending on the excitation, the behaviour of the system is significantly different. For the transverse electric wave case, *J*_*z*_ current only will be generated, and the electric quadrupole terms degenerate into magnetic dipole moments *m*_*x*_ and *m*_*y*_ that, in addition to the pure magnetic dipole terms, contribute to the scattered field. For highly tailored electric, magnetic dipole interactions, these quadrupolar responses are an unwanted complication as they produce varying degrees interference effects between sharp quadrupolar resonances and broader dipole modes^12^.

## Electronic supplementary material

Supplementary Information

Simulated logarithmic scaled power flow time average arrow plots recorded from 5 to 25 GHz.

Simulated logarithmic scaled power flow time average arrow plots recorded for a 0 to 45-degree incident plane wave.

Simulated surface magnetic field amplitude plots normalised to free space recorded from 5 to 25 GHz.

Simulated logarithmic scaled surface arrow & streamlines for the x and y magnetic field components recorded from 5 to 25 GHz.

Simulated surface magnetic field amplitude plots normalised to free space recorded for a 0 to 45-degree incident plane wave.

Simulated logarithmic scaled surface arrow & streamlines for the x and y magnetic field components recorded for a 0 to 45-degree incident plane wave.
