# Supplementary material for: Printable all-dielectric water-based absorber
Source: Sci Rep. 2018 Sep 27;8:14490. doi: 10.1038/s41598-018-32395-1 (PMC6160485; doi:10.1038/s41598-018-32395-1)
Supplement: Supplementary file 1 — Supplementary Information [file 41598_2018_32395_MOESM1_ESM.pdf]

# Printable all-dielectric water-based absorber

**Patrick J. Bradley[1,2*], Max O. Munoz Torrico[1], Conor Brennan[2], and Yang Hao[1]**

[1]School of Electronic Engineering and Computer Science, Queen Mary University of London, London, E1 4NS, UK
[2]School of Electronic Engineering, Dublin City University, Dublin, D09 W6Y4, Ireland
[*]p.bradley@qmul.ac.uk; y.hao@qmul.ac.uk

## Supplementary Information

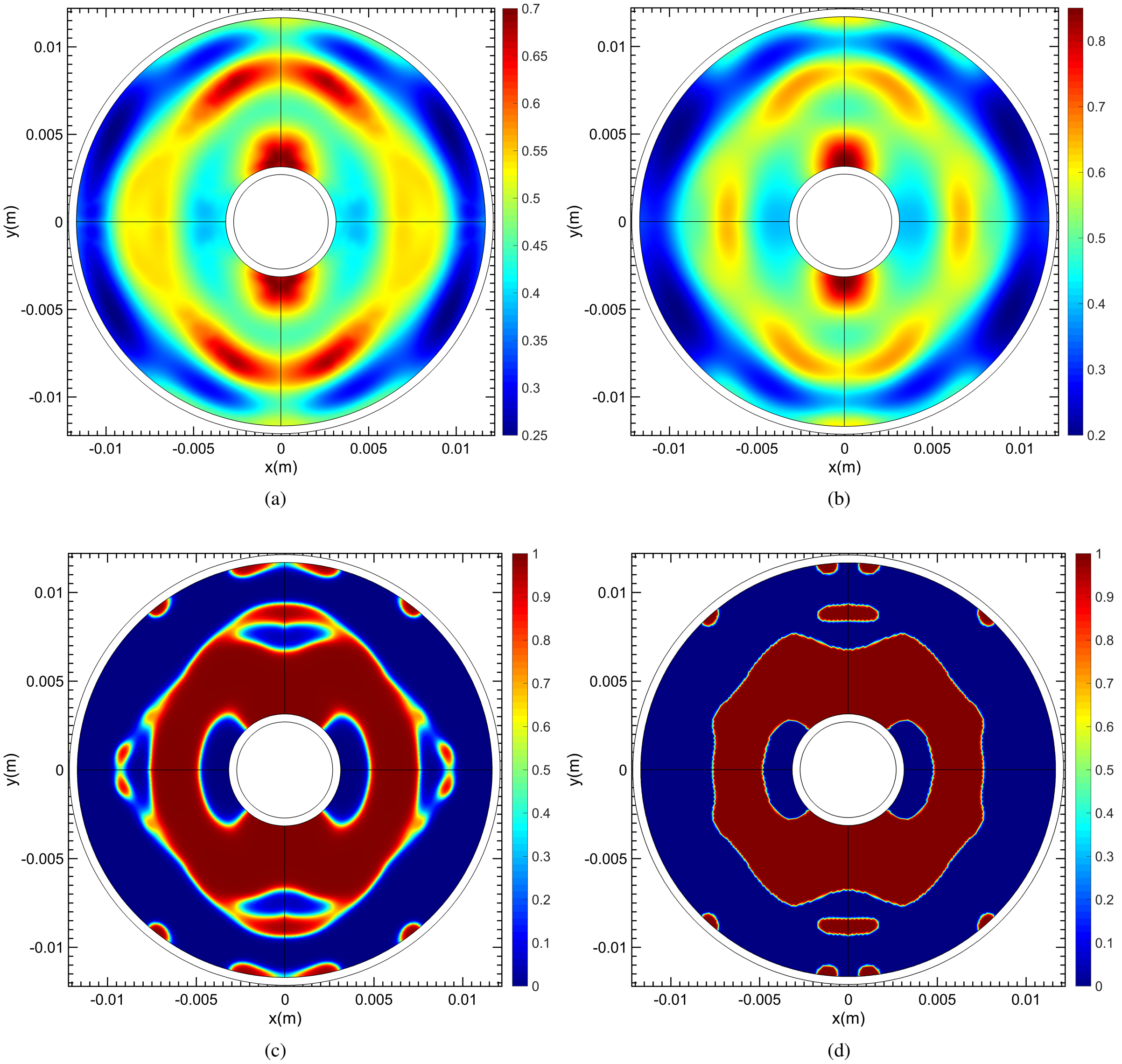


**Figure 1. Projected filtered density value $\overline{\rho}(\rho)$ at 4 discrete iteration cycles, where unity indicate a seed permittivity (water) and zero, the parent (VeroClear). a**, Iteration 100 **b**, 200 **c**, 400 **d**, final iteration 600 with a final seed volume fraction of 38%.

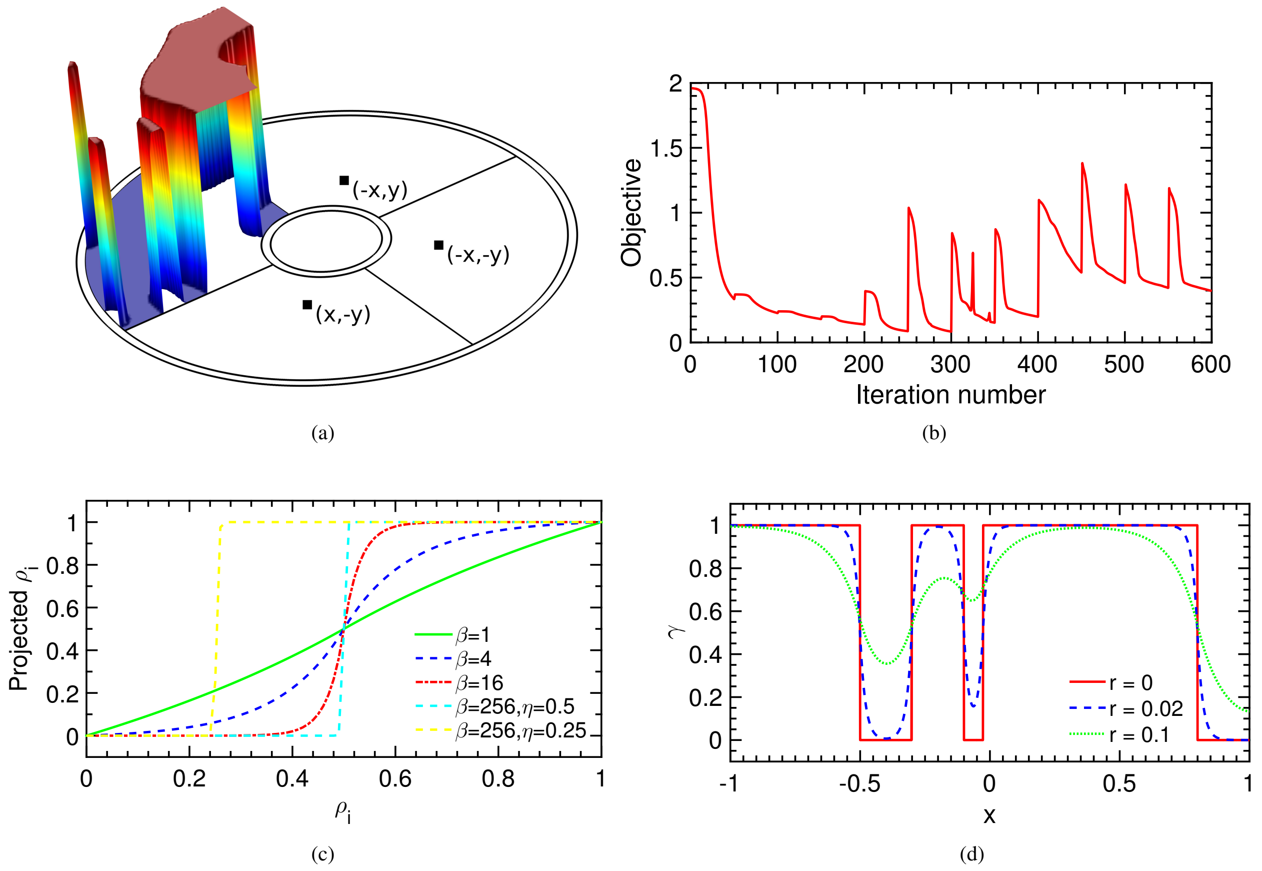


**Figure 2. Topology optimisation convergence and control parameters plots. a**, projected filtered density value $\tilde{\rho}$ at final iteration for a single quadrant, which undergo a 90, 180 and 270 degree clockwise rotation transformation ensuring symmetric and angular independent absorption. **b**, convergence plot of optimisation objective function, manifestation of doubling the projection parameter $\beta$ every $50^{th}$ iteration is evident **c** the projected physical density function controlled by $\beta$ to ensure smooth convergence by a controlled projection of the filtered density value $\widetilde{\rho}$ to a binary solution **d**, and Helmholtz PDE density filter example for 1D input comparing effect of different length parameters $r$.

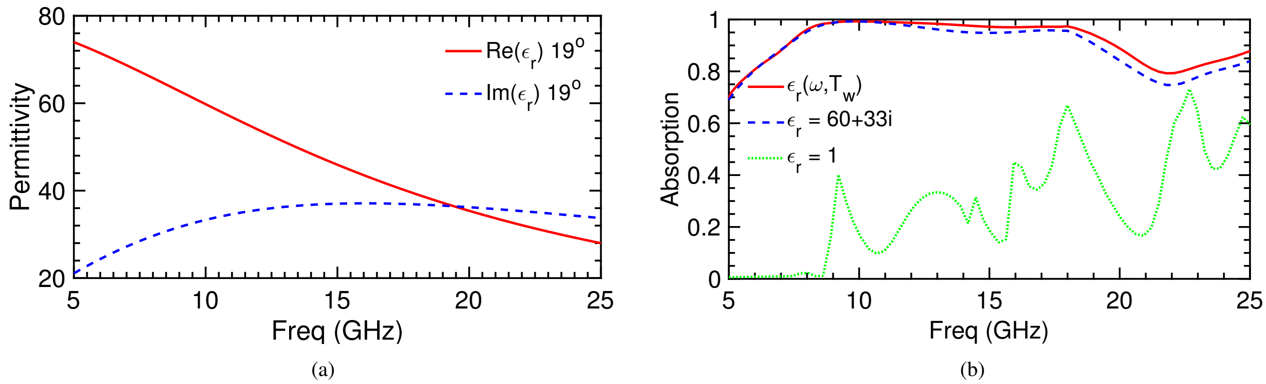


**Figure 3. Dielectric profile water and absorption as a function of permittivity. a**, thermal and frequency dependent profile of water permittivity for $19^o$ as used in the optimisation sequence and as defined in the Method section. **b**, absorption as a function of seed permittivity comparing water (**red**) as defined above vs an averaged uniform permittivity of $\varepsilon_r = 60 + 33\imath$ (**blue**) with that of replacing water with air (**green**), indicating switchability of our device from absorption to transmission.

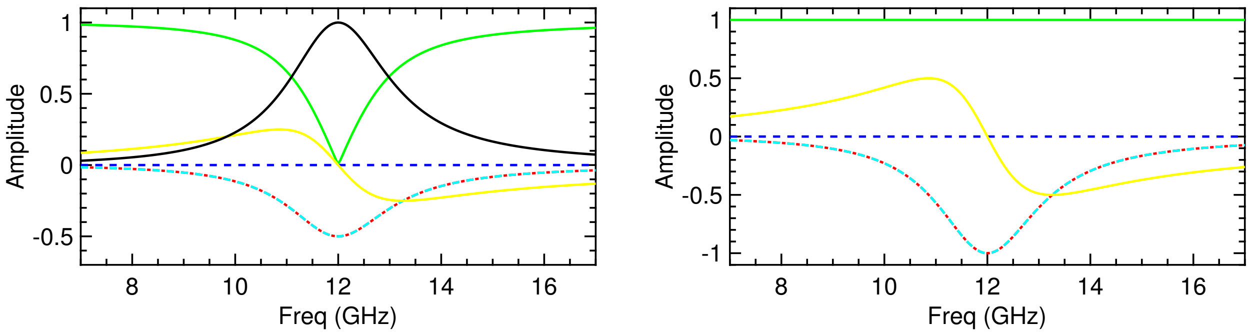


(a) (b)

**Figure 4. Analytical results for spectral overlapping electric and magnetic resonance of equal strength and width for an ideal loss-less metamaterial, demonstrating the effect of changing the magnitude of these resonances in tandem and extracted theoretical electric and magnetic dipole responses. a**, consequence of real part of the electric (**red**) and magnetic (**cyan**) resonances being equal to negative half of the incident field amplitude, therein, we observe a flat line reflection (**blue**) with zero transmission (**green**) and perfect absorption (**black**) of unity. **b**, correspondingly, if we double the electric (**red**) and magnetic (**cyan**) resonances we move from perfect absorption at a single frequency to perfect unity transmission (**green**).

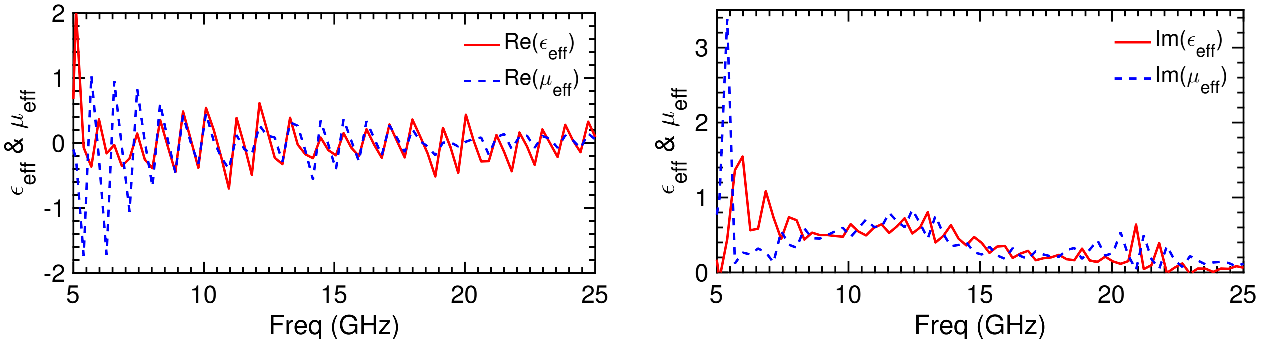


(a) (b)

**Figure 5. Extracted effective material coefficients from simulated S-parameters for normally incident plane wave. a**, real part of the inverted effective permittivity (**red**) and permeability (**blue**) **b**, related imaginary components.

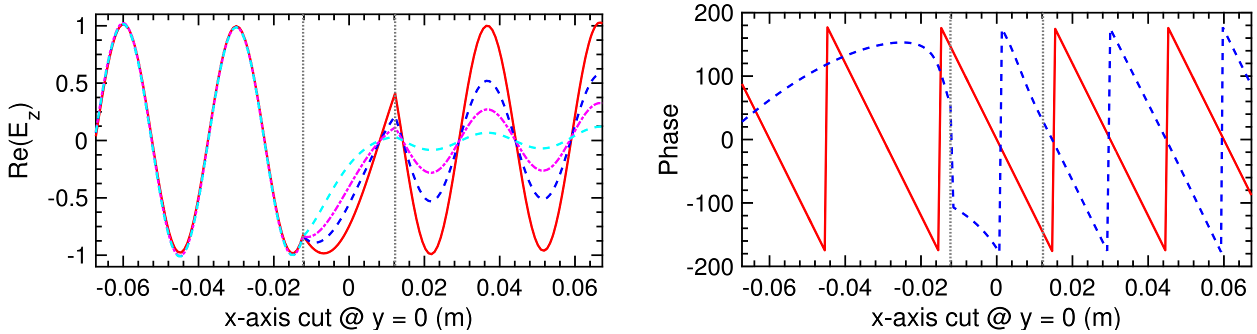


(a) (b)

**Figure 6. Line cut of electric field along the x-axis @ y=0 where the cylindrical array has been replaced with the equivalent constant medium. a** normalized electric field values for 4 difference vales of loss for an equivalent homogeneous medium with thickness 24.45 mm, where grey lines indicate boundary of the medium at 10 GHz, $n = -0.5 + 0.5\imath$ (**cyan** - averaged extracted refractive index), $n = -0.5 + 0\imath$ (**red**), $-0.5 + 0.125\imath$ (**blue**) and $-0.5 + 2\imath$ (**magenta**). This plot illustrates the conditions required to observe a near-unity incident and near-zero transmission along the x-axis @ y=0. **b**, associated phase of incident (**red**) and scattered electric field (**blue**) for same equivalent media with $n = -0.5 + 0.5\imath$ at 10 GHz. In this arrangement, destructive interference between the two waves of equal amplitude but $\pi$-path delay take place in the forward direction, resulting in cancellation of the total electric field ensuring zero transmission as predicted by theory

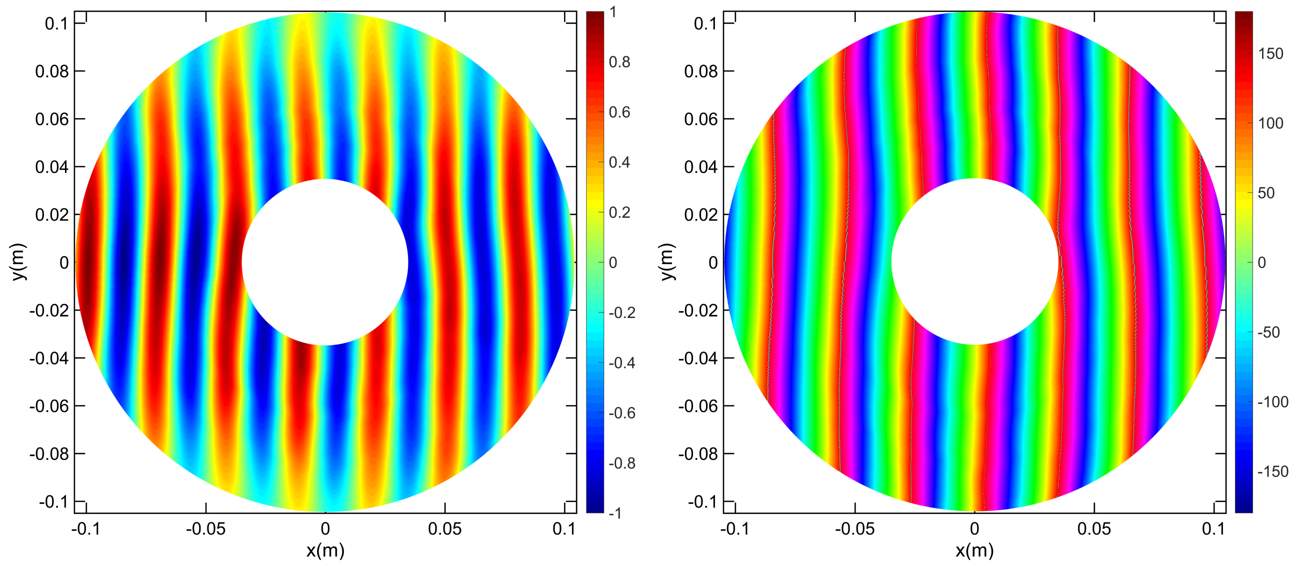


(a) (b)

**Figure 7. Normalised free space measurements probed within a parallel plate waveguide chamber at 10GHz which facilitate the normalisation of all subsequent measured electric fields to the free space. a**, measured real component **b**, and corresponding phase of the total electric field. Measurements were probed within a 5mm radial, $0.25^o$ angular resolution and interpolated using a linear function. The chamber was excited by a rectangular x-band waveguide and converted to a normally incident plane wave by a cylindrical-to-plane wave transformer lens as described in Method section.

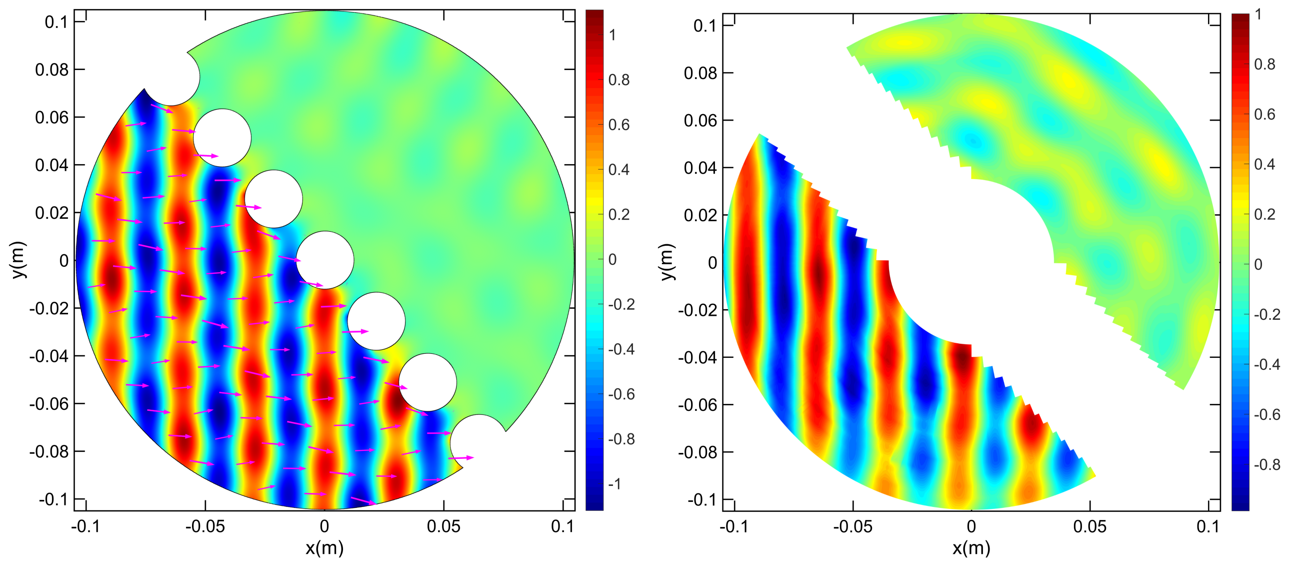


(a) (b)

**Figure 8. Simulated and measured real component of total electric field, z component (V/m), normalised to free space and power flow time average arrow plot at varying frequencies for a** $45^o$ **incident plane wave excitation at 10 GHz with 7 cylindrical absorbers periodical arranged along the y-axis @ x = 0. a**, simulated electric field and power flow time average arrow plot **b**, associated measured results for same set-up.

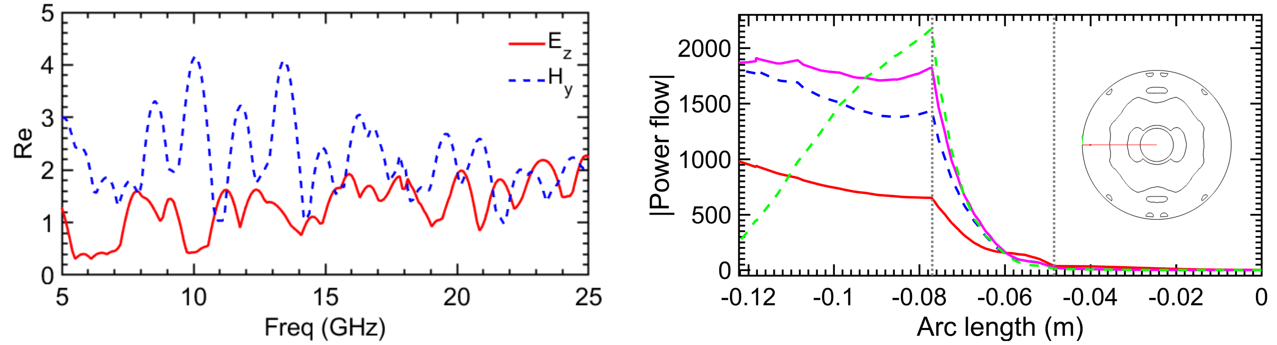


(a) (b)

**Figure 9. Simulated results for the surface electric field, magnetic field and power flow time average within the cylinder design boundary. a**, real component of the electric in z (**red**) and magnetic in y (**blue**) surface maximum values normalised to free space, which illustrate the complexity of the electric and magnetic enhancements across the frequency spectrum of interest. **b**, simulated power flow time average x component extracted from along the x-axis @ y=0 as per insert, where grey lines indicate boundary of the water medium recorded at key electric and magnetic surface maxima's, 7.82 (**red** 1), 12.37 (**magenta** 2), 13.48 (**blue** 3) and 16.31 (**green** 4) GHz.

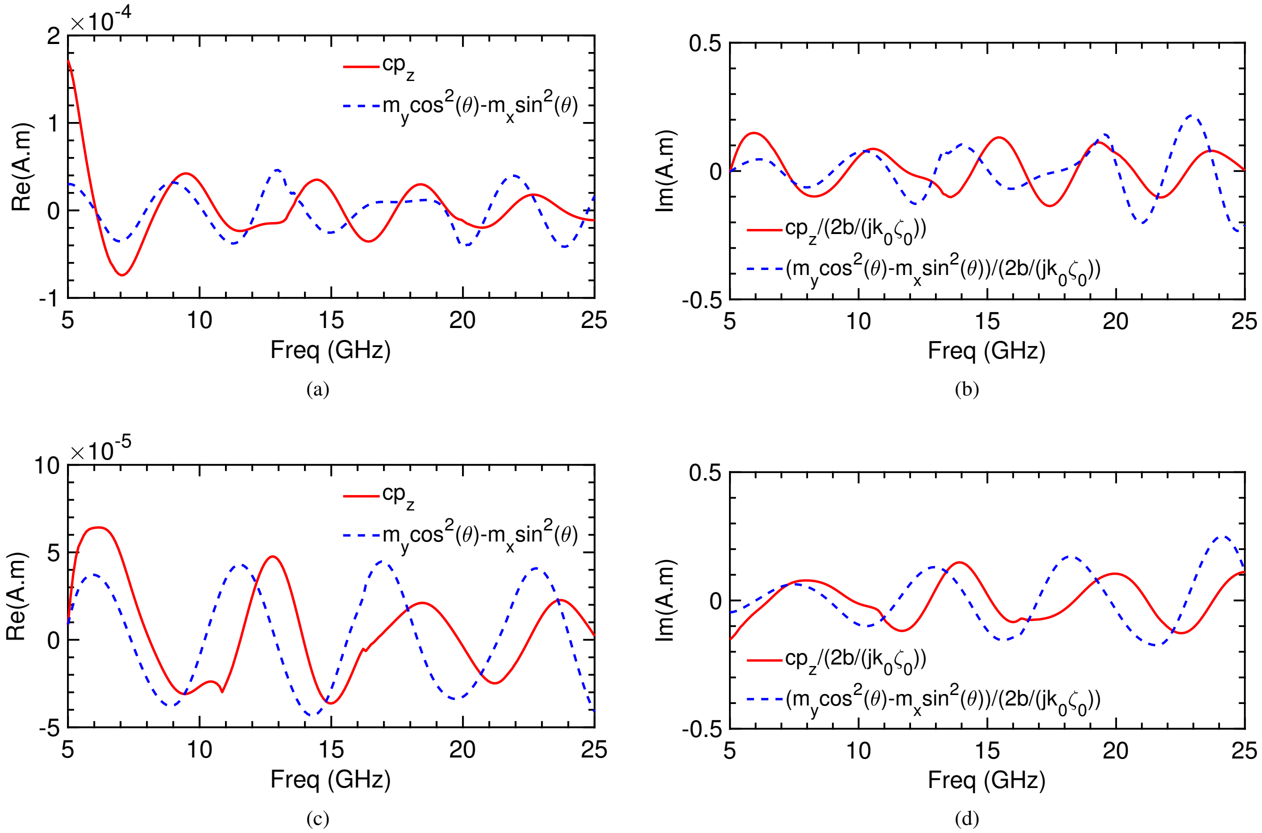


**Figure 10. Simulated extracted equivalent moment for absorber design subject to a 20 and 40 degree plane wave incidence. a,c**, Real and **b,d**, scaled imaginary component of the electric (**red**) and magnetic (**blue**) dipole moment resonances for 20 and 40 degree incidence respectively, where a zero response fulfils the theoretical case for perfect absorption.

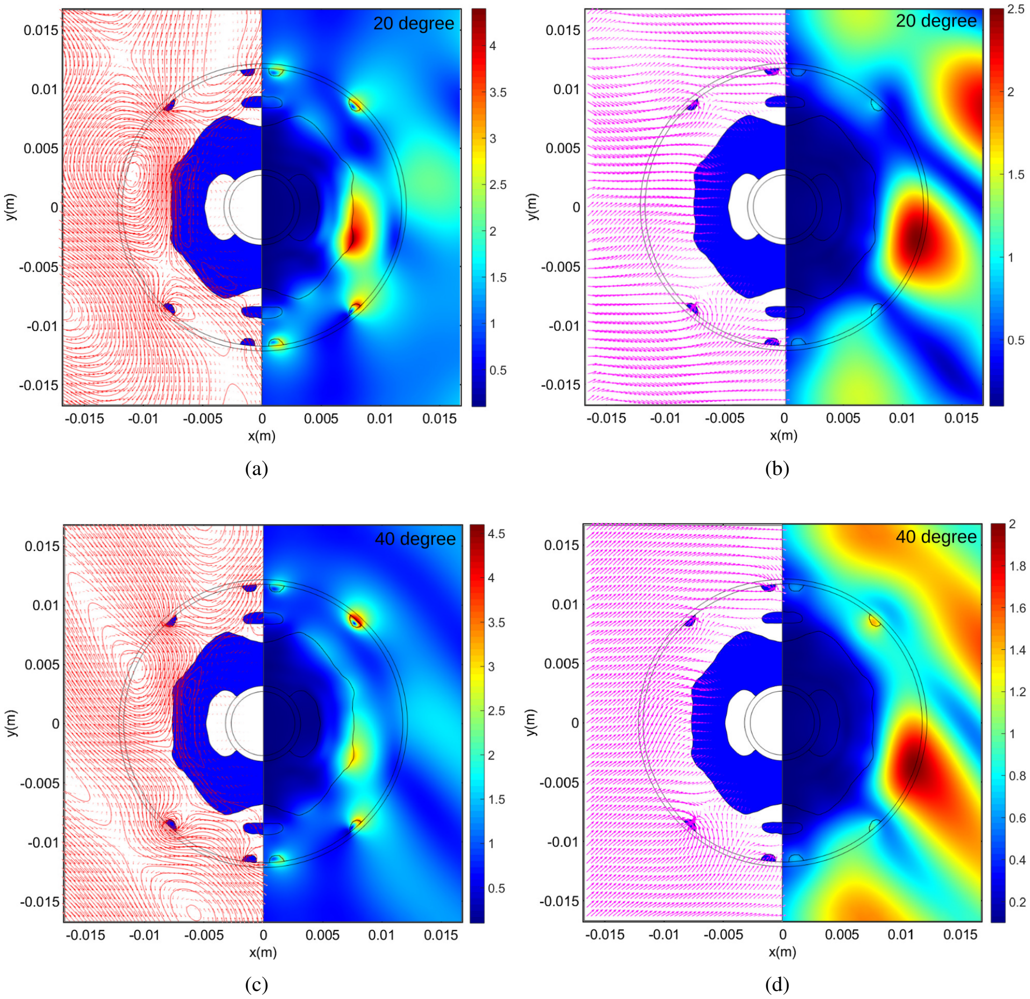


**Figure 11. Simulated surface magnetic, power flow time average and electric field amplitude plots normalised to free space at 13.48 GHz for two discrete incident angles. a,c** -x illustrates logarithmic scaled surface arrow & streamlines for the $x$ and $y$ magnetic field components at 20 and 40-degree illumination indent from LH boundary, +x $180^o$ rotated image of the corresponding magnetic field magnitude. **b,d** -x illustrates related logarithmic scaled power flow time average arrow and +x the $180^o$ rotated electric field magnitudes. As the incident field rotates there is a corresponding shift in the magnetic modes orientation. However, as expected, the magnetic field magnitude remains nearly unchanged from normal incidence due to the balancing of $y$ magnetic components with its $x$ contribution. Together with Figs. 10(a)-10(d) these plots provide valuable insight into the nature of the angular stability.
