# Supplementary figures and images for: Printable all-dielectric water-based absorber
Source: Sci Rep. 2018 Sep 27;8:14490. doi: 10.1038/s41598-018-32395-1 (PMC6160485; doi:10.1038/s41598-018-32395-1)

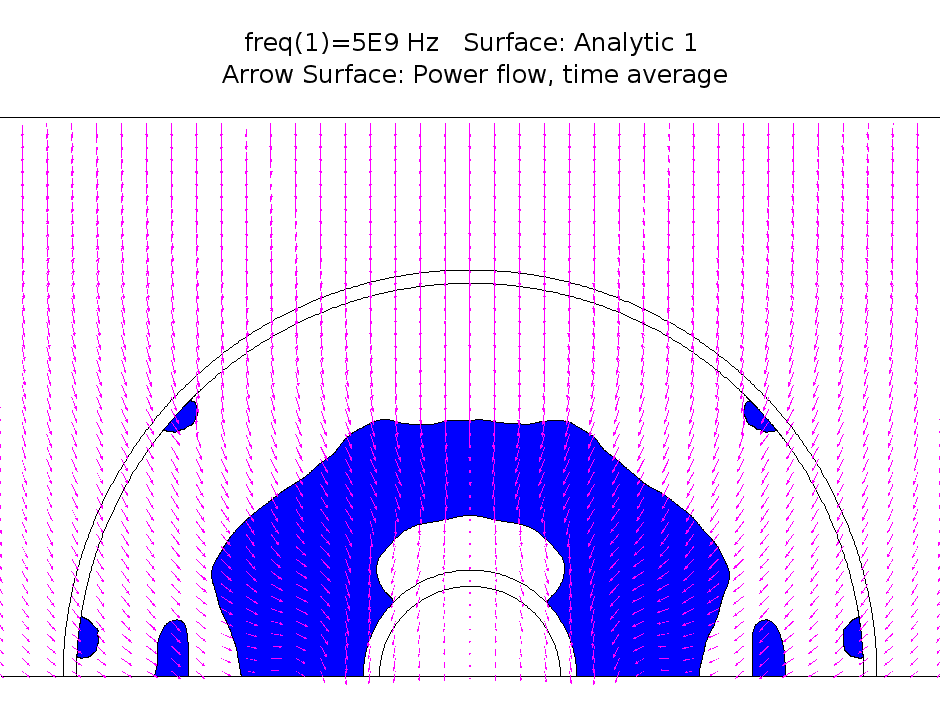

Supplement: Supplementary file 2 — Simulated logarithmic scaled power flow time average arrow plots recorded from 5 to 25 GHz. [file 41598_2018_32395_MOESM2_ESM.gif]

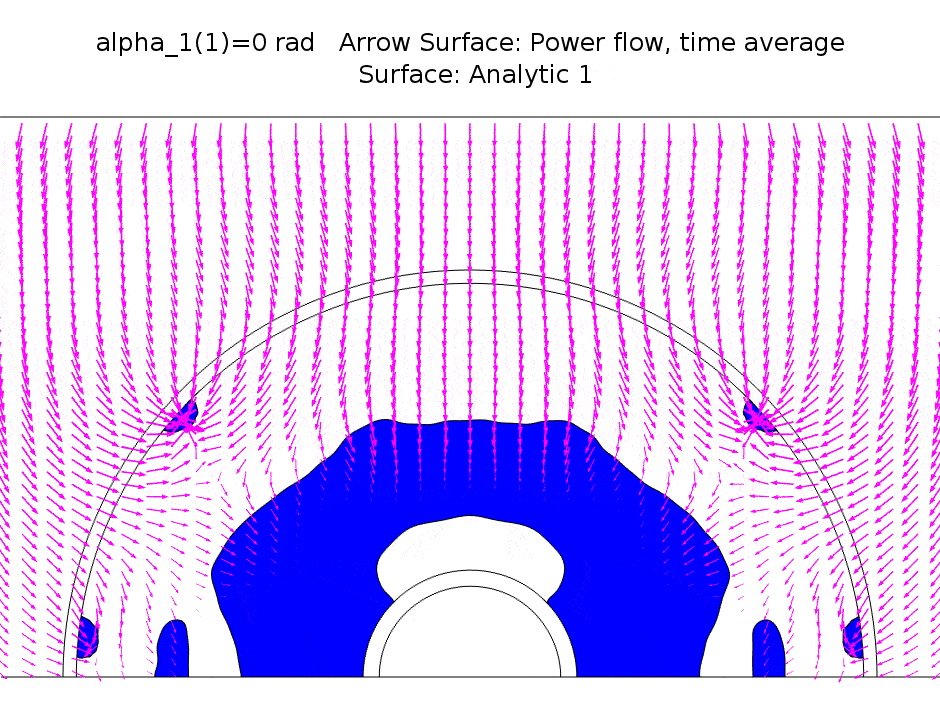

Supplement: Supplementary file 3 — Simulated logarithmic scaled power flow time average arrow plots recorded for a 0 to 45-degree incident plane wave. [file 41598_2018_32395_MOESM3_ESM.gif]

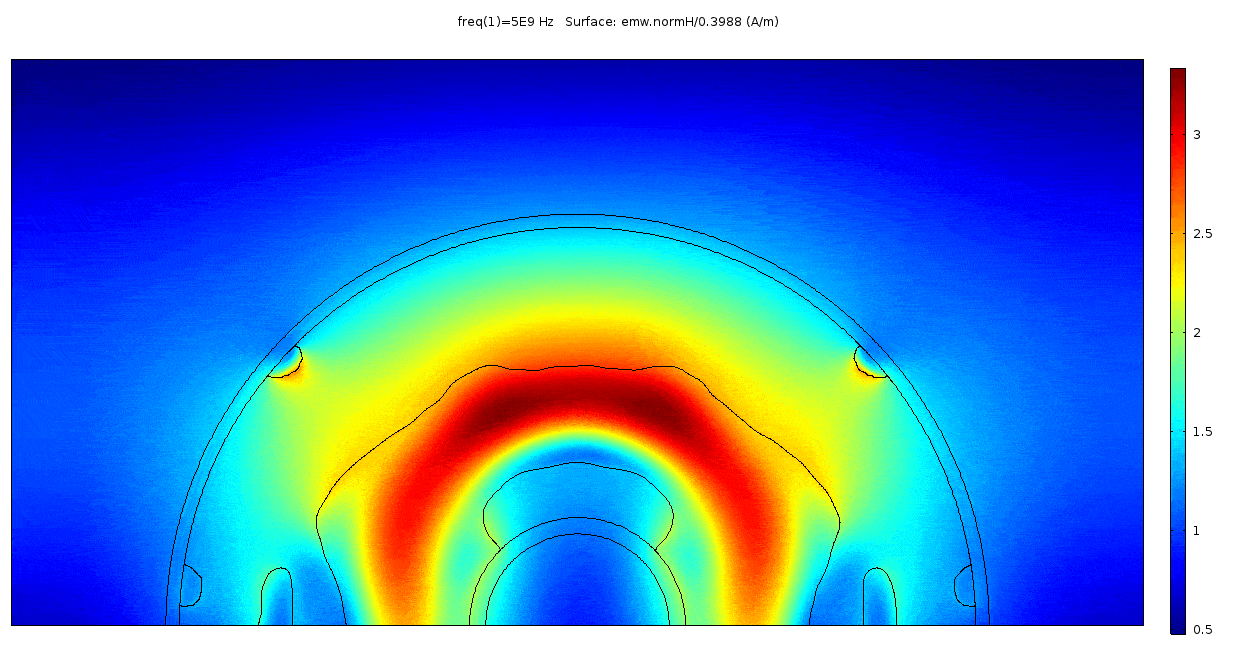

Supplement: Supplementary file 4 — Simulated surface magnetic field amplitude plots normalised to free space recorded from 5 to 25 GHz. [file 41598_2018_32395_MOESM4_ESM.gif]

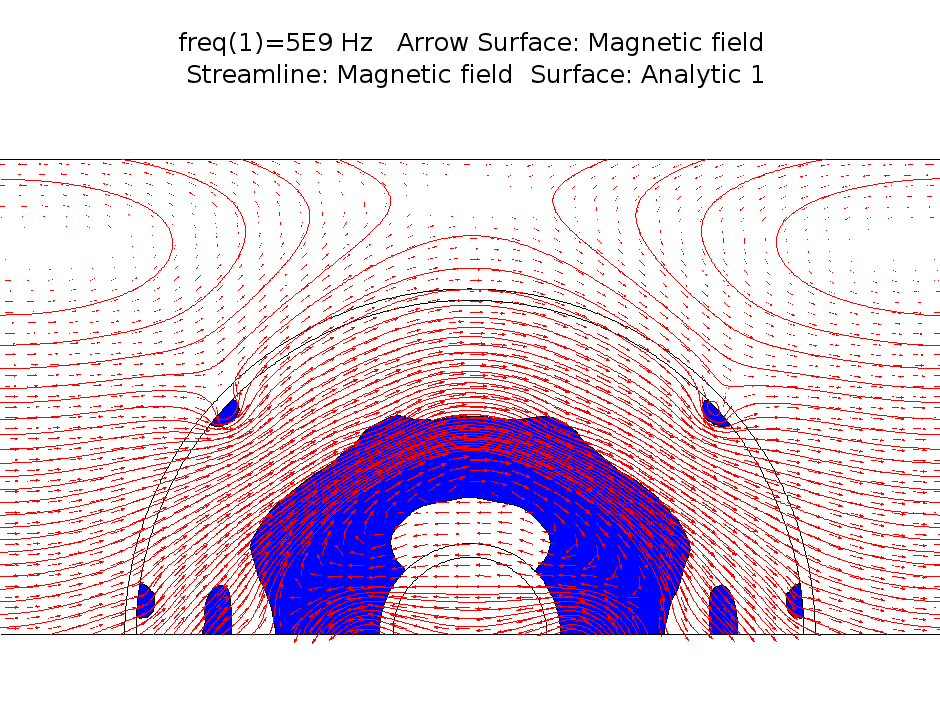

Supplement: Supplementary file 5 — Simulated logarithmic scaled surface arrow & streamlines for the x and y magnetic field components recorded from 5 to 25 GHz. [file 41598_2018_32395_MOESM5_ESM.gif]

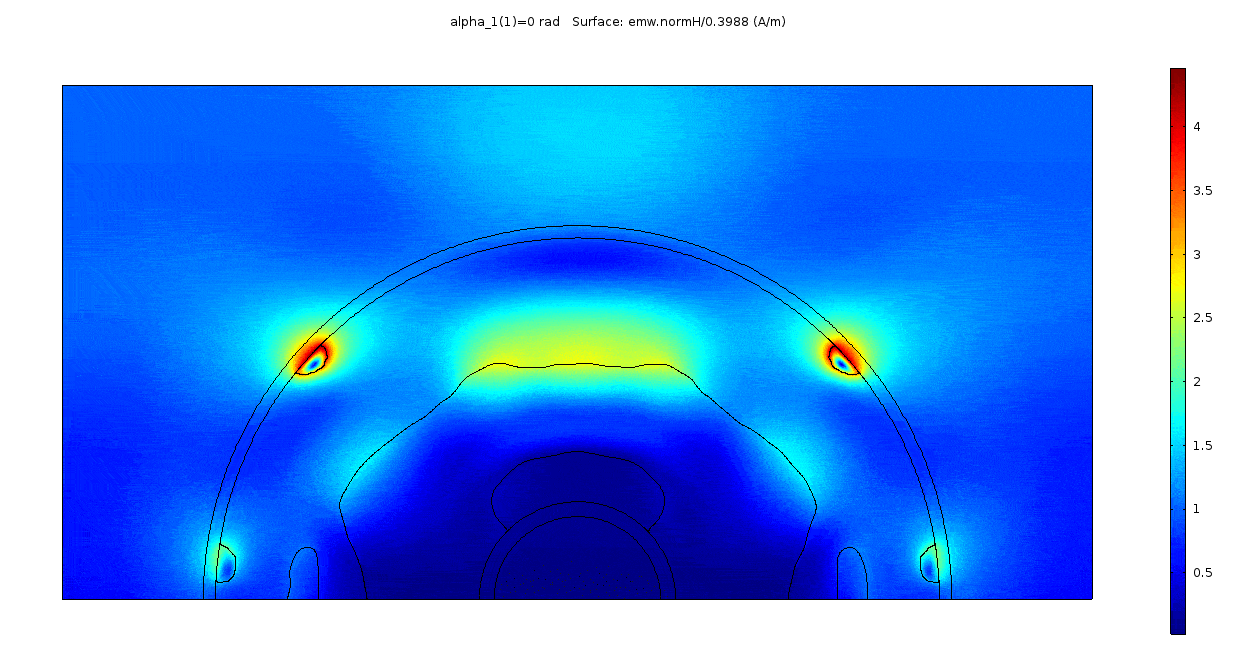

Supplement: Supplementary file 6 — Simulated surface magnetic field amplitude plots normalised to free space recorded for a 0 to 45-degree incident plane wave. [file 41598_2018_32395_MOESM6_ESM.gif]

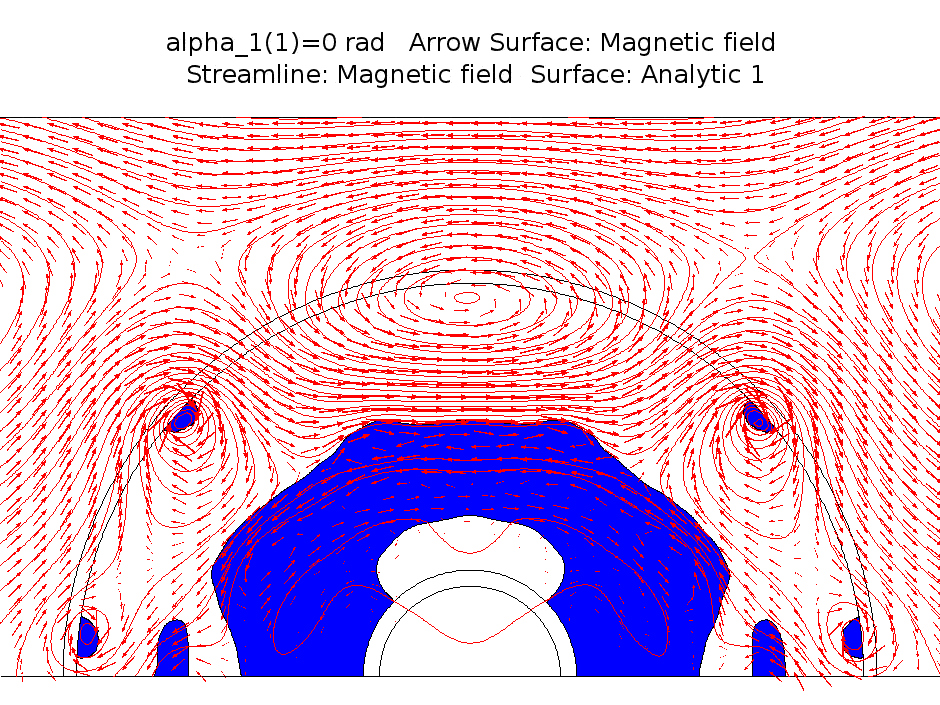

Supplement: Supplementary file 7 — Simulated logarithmic scaled surface arrow & streamlines for the x and y magnetic field components recorded for a 0 to 45-degree incident plane wave. [file 41598_2018_32395_MOESM7_ESM.gif]
